# Visual evaluation of sliced Italian salami by image analysis

**DOI:** 10.1002/fsn3.540

**Published:** 2017-11-12

**Authors:** Annalisa Romano, Paolo Masi, Silvana Cavella

**Affiliations:** ^1^ Centre for Food Innovation and Development in the Food Industry Portici (Naples) Italy; ^2^ Dipartimento di Agraria University of Naples Federico II Portici (Naples) Italy

**Keywords:** Fat content, image analysis, meat, pork, sliced Italian salami, structure

## Abstract

Visual inspection is an important part of quality control not only for manufacturers but also for retailers and consumers. The object of this investigation was to determine fat content in sliced salami by means of image analysis. The image analysis procedure is applied to digital images of sliced Italian salami produced in 16 different salami factories (A–P). The image analysis method described in this work is nondestructive and the necessary equipment is cheap. It extracts directly interpretable parameters of fat particle morphology (e.g., area, roundness) and number of fat particles from 15 digital images for each sample (A–P). The correlations between the fat features extracted from the images with the chemical fat content measured on the samples were also studied. Good relationships were found between the fat particle characteristics measured by image analysis procedure and the percentage of chemically extractable fat by correlation (*R* ^2^=0.75) and principal component analysis.

## 1 INTRODUCTION

Dry fermented sausages are meat products with a high‐fat content, which is visible when the product is sliced. Among dry fermented sausages, approximately one hundred varieties of Italian salami have been described, even if many of them are not industrial products (Conter et al., 2007). The wide variety of dry fermented sausages in Italy is due to variations in raw material, formulation, and manufacturing processes which come from the traditional habits of different regions. Usually, Italian salami are cylinder‐shaped, weigh from 200 to 2,500 g, have a diameter of about 2–10 cm and a length ranging from 10 to 50 cm. They are generally prepared of coarsely minced lean pork, mixed with fat similarly cut into small pieces, with the addition of pepper, salt, and skimmed milk powder.

The chemical composition, fat content, and visual texture of meat based products, for example, salami, have a large impact on their sensory quality and commercial value. In particular, appearance, meant as color and visible fat content, is a very important criterion in choosing and buying meat and meat products (Dransfield et al., 2005; Fortomaris et al., 2006). Sliced salami is mostly packaged in modified atmospheres and displayed under light either in chill cabinets or at room temperatures (Sørheim, Måge, & Larsen, 2017). They are widely produced in Europe and other parts of the world. The ratio of visible fat in salami is an important discriminating factor on purchase because this product usually has a high‐fat content (Girolami, Napolitano, Faraone, Di Bello, & Braghieri, 2014). Previous studies have proved that juiciness and acceptance of the meat is largely influenced by its fat content in calf (Penfield, Costello, McNeil, & Rienmannn, 1989), lambmeat (Touraine, Vigneror, Touraille, & Touraille, 1984), beef (Du, Sun, Jackman, & Allen, 2008; Hur, Park, & Joo, 2008) and pork (Fernàndez, Monin, Talmant, Mourot, & Lebret, 1999; AMSA, 2001; Mòrlein, Rosner, Brand, Jenderka, & Wicke, 2005; Fongaro et al., 2015).

One of the proved methods for objective assessment of the fat level is chemical analysis. The AOAC protocol (1991) is commonly used to extract and quantify the fat content by ether. Such method is reliable, but slow, tedious, relatively costly, destructive, environmentally harmful, and unable to provide the information of size and spatial distribution of fat particles. Although the total quantity of fat is important, the size and distribution may also be relevant with meat quality (Ferguson, 2004). In fact, the appearance of products unities is a primary criterion in making purchasing decisions (Kays, 1991). Consumers select their foods in the supermarket primarily based on visual perception and often this is the only direct information received from the product. Digital vision systems are a very current choice for delivering fast, reliable, and robust food classification as the grading of foodstuffs by human graders has essential weaknesses of subjectivity, inconsistency, and unreliability (Jackman & Sun, 2013). Recent advances in the area of computers and image processing have generated new ways to estimate the lipid content of meat and meat products. Numerous researchers have investigated the possibility of using image‐based meat quality evaluation, and a number of reviews have listed the main applications for food and more specifically meat science (Damez & Clerjon, 2008; Jackman & Sun, 2013; Wu & Sun, 2013).

Artificial vision consists of associating a camera, devoted to image acquisition, with a computer used for image analysis. The image analysis is implemented to characterize an object or a surface on the visualized image, in particular by analyzing the texture. The image analysis is a nondestructive procedure which has been in use for some years in the meat industry to evaluate meat quality, including color measurement of pork meat (Chmiel, Słowiński, & Dasiewicz, 2011; O'Sullivan et al., 2003) and the content of intramuscular fat in pork meat and products (Faucitano, Huff, Teuscher, Gariepy, & Wegner, 2005; Mendoza, Valous, Sun, & Allen, 2009). Although studies of the characterization of fat in the meat products applying The image analysis technique is gaining interest in scientific literature (Serrano, Perán, Jiménez‐Hornero, & Gutiérrez de Ravé, 2013), the automation of computer vision system for industrial practice still presents several challenges, for example, standardization (Tan, 2004). In fact there is not yet a standardized method for these evaluations as differences among the reported image analysis methodologies are found. An accurate observation technique of the shapes and the sizes of fat particles, and their distribution are thus required.

The object of this study was to present a cheap and analytical protocol of image analysis to extract directly interpretable parameters of fat content of sliced salami, such as area slice salami, fat size (e.g., area), shape descriptor (fat roundness), count of fat particles and total fat area per unit area and distribution of fat particles of salami.

The performance of the proposed protocol was verified by correlation analysis between the visible fat content obtained by digital system and the percentage of chemically extractable fat content of sliced salami.

## 2 MATERIALS AND METHODS

### 2.1 Samples

The study was carried out on 48 commercial Italian dry‐ cured salami (2.5 kg each) produced by 16 different Italian manufacturers (A–P). Three dry‐cured salami were kindly provided by each manufacturer (A–P).

Commercial dry‐cured salami were consisted of coarse mixtures of pork meat and fatty tissues (lard) combined with salts, nitrite (curing agent) and spices as nonmeat ingredients. All samples were produced in four main steps: traditional smoking, fermentation, slow drying and ripening. Smoking was conducted in smoking chambers at a temperature of 20–35°C and a relative humidity of 80%–90%, for a period of 3 days. Drying was done in drying chambers with controlled microclimatic conditions (air circulation—10–20 cm/s; temperature—12–16°C; humidity gradually reduced from 90% to 80%) lasting 10 days. Ripening of salami took place in cellars with a stable microclimate and an air temperature which does not exceed 15°C and relative air humidity between 65% and 80% until they become 40 days old.

Three different test production runs were obtained from each manufacturer (A–P) at the end of ripening (3 independent production runs×16 manufacturers). All Italian salami (48) were chilled to 4°C before slicing. Salami were cut at 20°C using an electrical knife. Five salami slices (thickness=2 mm) were taken from the middle of each sample (5 slices×3 independent production runs×16 manufacturers).

The chemical properties indicated by the producers were: pH≥4.8; humidity (%) between 25% and 45%; protein ≥15.0% and ash ≥4.5.

### 2.2 Image analysis

The salami slices were photographed by means of a digital camera (Olympus^®^ C‐7070Wide ZOOM, Milan, Italy) placed on the top of a black box with four light lamps (LED G13, Philips, Italy) at standardized conditions (20 W, 3,000 K, fixed light distribution). The distance between the light source and the bottom plate was fixed (45 cm).

Fifteen 2D images for each salami samples (A–P) were acquired immediately after slicing for a total of 240 images. The images were processed by Image Pro Plus 6.1 software (Media Cybernetics Inc.) using the method described by Romano, Cavella, Toraldo, and Masi (2013) with some modifications. Briefly the RGB images were recorded and converted to a 256 level gray scale. A contrast enhancement procedure, some filters and dilation/erosion operations were applied to better discriminate fat particles (“objects”: white section) from images (“meat cells or matrix”: red section). The number of white and black pixels was calculated for each salami slice. White objects correspond to the fat particles and dark objects correspond to the meat matrix. Computed parameters included the following: number of fat particles counted (*n*); area salami slice (*A*_s_); fat particle area (AF_*i*_); fat particle roundness (RF_*i*_):

(1) $${\text{RF}}_{i}=\frac{P_{i}^{2}}{4\cdot \pi \cdot {\text{AF}}_{i}}$$

where AF_*i*_ is the area of the *i* ^th^ fat particle and *P*_*i*_ is the measured perimeter of the *i*th fat particle. The roundness calculates the circularity of a fat particle. Theoretically it is 1 for a perfect circle while approaches to zero for a line.

Based on these parameters, the visible fat content (V FC) was computed as:

(2) $$\text{Visible fat content}\left(\%\right)=\frac{\sum_{i}^{n}{\text{AF}}_{i}}{A_{s}}\times 100$$

Samples are characterized and classified by statistical parameters (arithmetic mean and maximum value) of area ($\bar{{\text{AF}}_{i}}$ and AF_*i*max_) and roundness ($\bar{{\text{RF}}_{i}}$ and RF_*i*max_). Each result is the average of fifteen measurements (5 slices×3 batches).

### 2.3 Chemical analysis

Soxhlet extractions were carried out using a Büchi B‐811 Soxhlet apparatus (Büchi Labortechnik, Flawil, Switzerland) according to AOCS Method Ba 3– 38 (American Oil Chemists’ Society, 1998). Five salami slices were blended using a blender (LB20ES, Torrington, CT). Total fat amount of samples was evaluated on a gravimetric basis and expressed as percent by weight. Average values of three measurements were calculated for each sample.

### 2.4 Statistical analyses

Statistical analyses were performed using SPSS version 19.0 (SPSS Inc., Chicago, IL). One way (ANOVA) and post hoc (Duncan test) test were performed to determine the statistical significance. The confidence interval was 95% (*p*≤.05). Furthermore, a principal component analysis (PCA) was performed to interpret visual attributes and chemical parameter in salami. To decide the number of principal components (PCs), the eigenvalues of the correlation matrix, indicating the percentage of variability explained by each component, were tabulated and a scree plot was constructed.

## 3 RESULTS AND DISCUSSION

### 3.1 Image analysis

**Figure 1:**
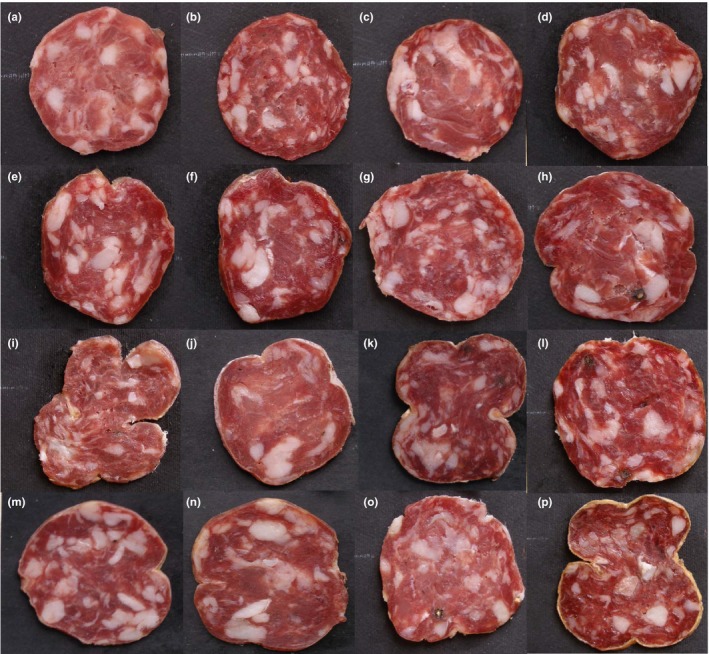
Image of sliced salami batches a–p

Representative 2D images of salami samples are shown in Figure 1. Fat particles can be distinguished as white spots embedded in red meat. Although the salami were manufactured entirely from pork meat, the samples investigated in this study have not similar visual appearance due to visible fat content (Figure 1). The visible fat content was characteristic of each product (A–P trials).

The evaluated slices showed a great degree of fat heterogeneity, making its visual characterization a difficult task.

**Figure 2:**
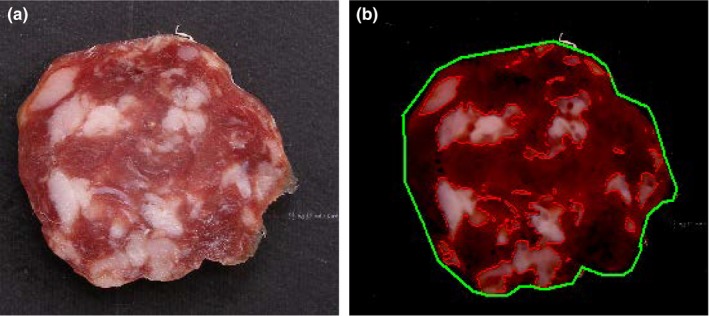
Image of salami slice (a) and processed image of salami slice (b)

The high performance (quality, contrast, number pixels) of images allowed a precise qualitative and quantitative analysis, but in order to discriminate and evaluate fat particles from salami slices, a digital image analysis procedure (Figure 2) must be carried out on images of samples (Figure 1a). It was hypothesized that physical parameters remain constant on the salami length, that is the salami slice was representative of a salami of infinite length. This approximation is very important because it reduce the 3D problem to a 2D, with great advantages in term of computational time.

2D picture (Figure 2a) of a slice from a salami was acquired, than processed following the image analysis protocol (Figure 2b). The image analysis protocol extracts directly parameters of fat particle morphology (area, roundness), number of fat particles and permits determine the distribution of fat particles of salami without limits of view spatial field.

**Figure 3:**
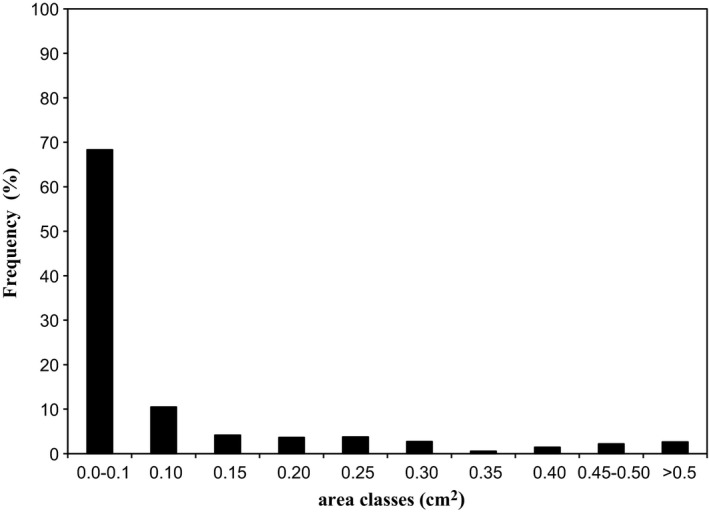
Typical frequency distribution of area of salami's FPs (Trial E)

The fat particle area distribution analysis was also carried out counting the percentages of fat particles falling into ten predefined area classes. In order to characterize the fat particle morphology of salami slice, the fat particle size distributions were calculated for all slices. Figure 3 shows for a representative sample (E) the frequency distribution of fat particles area. These statistical analyses provide supplementary evidence supporting the view that digital image can help in analyzing the appearance of fat particles in the salami slices. The image analysis procedure can detect very small particles, depending on the detection range of the software used to obtain results, here it recognizes both smaller (0–0.1 cm^2^) and larger fat particles (≥5 cm^2^). In particular, the lowest class of fat particle area (<0.1 cm^2^) represented the fat of pork meat, while fat particle area distribution between 0.1 and 0.5 cm^2^ was representative of fat added as an ingredient and it was due to both the random distribution of the fat particles during mixing by means of meat grinders and the deformation of fat particles during the operation of stuffing. The salami were packed into the casings as firmly as possible to avoid air pockets.

Results of the object analysis of fat particles are illustrated in Table 1. Samples can be characterized by count of fat particles and total fat area per unit area, statistical parameters of area (AF_*i*_) and roundness (RF_*i*_) of fat particles (Table 1). Significant differences (*p*<.05) were detected for total number of fat particles normalized by the total area of the salami slice which discriminated samples into five statistical groups (*p*<.05) and compared well with visual inspection of samples.

**Table 1:** Results after object analysis, expressed as means±SD

Samples	*n*/As	$\bar{{\text{AF}}_{i}}$ (cm^2^)	AF_*i*max_ (cm^2^)	$\bar{{\text{RF}}_{i}}$	RF_*i*max_	V FC (%)
A	1.44±0.2cd	0.085±0.01ab	0.81±0.5ab	1.50±0.5a	6.16±1.7bc	11.84±1.2b
B	1.10±0.2a	0.133±0.02fg	0.75±0.2a	1.79±0.3b	4.41±1.1ab	11.37±1.1b
C	0.99±0.2a	0.174±0.02e	1.25±0.0bc	2.03±0.3c	4.77±0.7ab	14.30±0.9cd
D	1.19±0.1ab	0.084±0.01a	0.83±0.4ab	1.72±0.0a	3.89±0.3a	8.74±1.0a
E	1.42±0.2c	0.083±0.00a	0.69±0.0a	1.90±0.1b	4.75±0.5ab	11.56±1.5b
F	1.30±0.1b	0.135±0.03fg	1.19±0.4bc	1.92±0.0bc	4.73±0.2ab	15.64±1.0d
G	1.38±0.2c	0.122±0.02ef	1.01±0.5b	1.93±0.1bc	4.48±0.5ab	14.11±1.1cd
H	1.54±0.6d	0.089±0.01b	1.19±0.3bc	2.08±0.1c	7.90±0.1d	11.33±0.2b
I	1.76±0.2e	0.075±0.01a	0.90±0.3ab	2.00±0.1c	6.41±0.0c	13.05±2.1bc
J	1.14±0.2ab	0.099±0.01bc	0.68±0.2a	1.86±0.2b	4.91±1.4b	9.75±0.6a
K	1.41±0.2c	0.112±0.01cd	1.60±0.2d	2.05±0.1c	6.18±0.5bc	15.60±1.5d
L	1.45±0.4cd	0.085±0.01ab	0.82±0.2ab	1.89±0.1b	5.53±1.3b	12.03±0.8b
M	1.40±0.2c	0.123±0.01ef	0.91±0.1ab	1.97±0.1c	5.22±0.3b	15.33±1.8d
N	1.29±0.1b	0.118±0.03de	1.03±0.2b	2.06±0.1c	5.71±1.2bc	14.44±0.7cd
O	1.87±0.2e	0.138±0.01g	1.33±0.3c	2.05±0.1c	6.32±1.1c	18.52±0.3e
P	1.68±0.4de	0.095±0.01c	1.20±0.3bc	2.08±0.1c	7.91±0.1d	14.46±1.6cd

Means with a column with different letters are significantly different (Duncan's test *p*<.05).

V FC, visible fat content.

The $\bar{{\text{AF}}_{i}}$ and AF_*i*max_, illustrated in Table 1, revealed that there were wide variations in the values for all examined samples. In particular, $\bar{{\text{AF}}_{i}}$ values were ranged between 0.075 and 0.138 cm^2^, while the AF_*i*max_ values between 0.69–1.33 cm^2^. Results were consistent with the used technology, in fact, pork fat was cut separately to a particle size of 3.5–5 mm and was then added to each production batch. The $\bar{{\text{AF}}_{i}}$ results were more discriminated than the AF_*i*max_ values, which separated samples into four statistical groups (*p*<.05).

Results of image analysis also showed significant differences (*p*≤.05) for $\bar{{\text{RF}}_{i}}$ and RF_*i*max_ of detected fat particles (Table 1). Roundness calculates circularity of a fat particle. $\bar{{\text{RF}}_{i}}$ of all samples were more than 1. Therefore RF_*i*max_ describes the maximum deviation of fat particles from a true circle. Variations of shapes show why particle extraction is not easy for digital image analysis and justifies the use of morphological treatment (erosion/dilation) in this study. The slice was in fact submitted to a dilation‐erosion with squared structuring elements, in order to close the matrix outlines. The $\bar{{\text{RF}}_{i}}$ ranged between 1.5–2.1. There was not a significant difference (*p*<.05) between the scores of the 50% of our samples. Results agree with visual characteristics of pork fat used as ingredient, which was cut by means of a meat grinder (10–12 mm). The highest RF_*i*max_ values (*p*<.05) were recorded for the H and P salami slices (7.9). The dissimilarities among samples were mainly due to fat percentage and size and any processing conditions which include duration of mechanical treatments (mixing and stuffing), since the raw material (pork meat) and conditions of smoking and ripening were the same.

The V FC of slice was estimated as the percentage fraction of the area of fat particles versus the total area of the salami slices (Table 1). Statistically significant differences were found among the different samples studied (*p*<.05).

### 3.2 Correlation analysis

**Figure 4:**
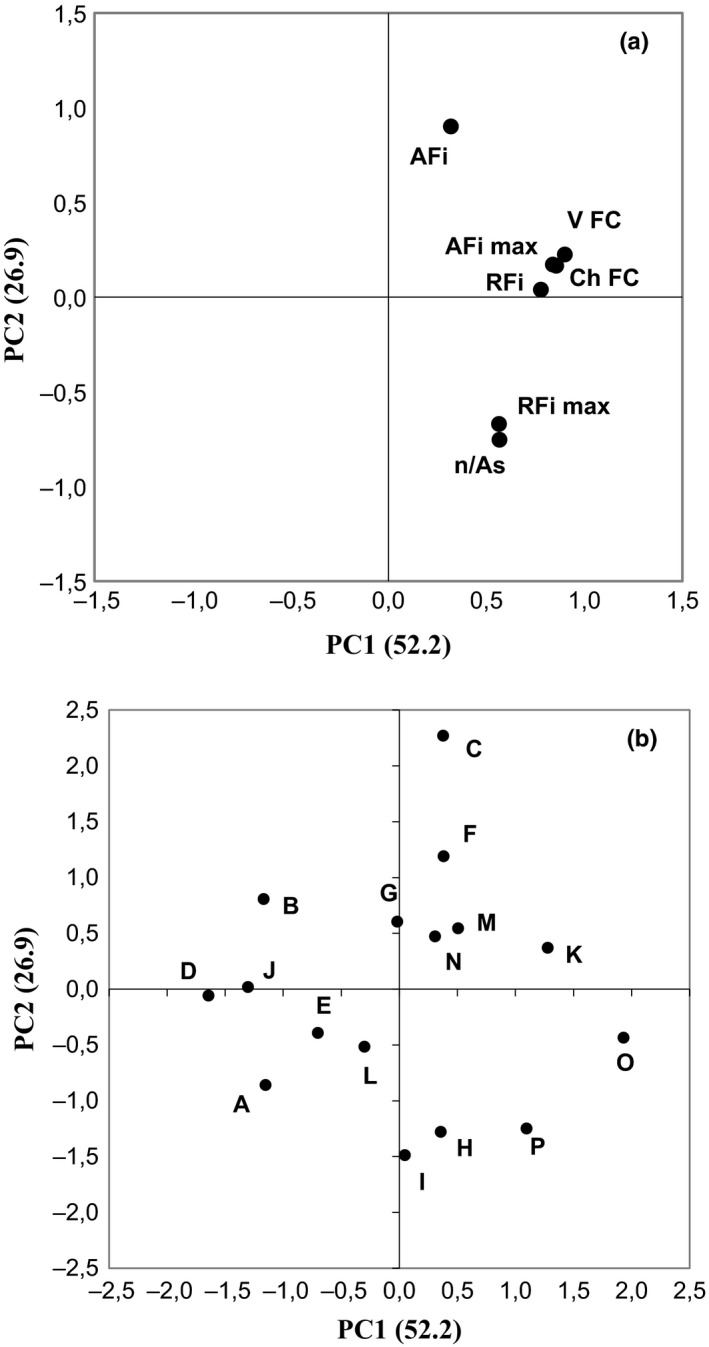
Results of principal component analysis—loading and score biplot for A–P samples

**Figure 5:**
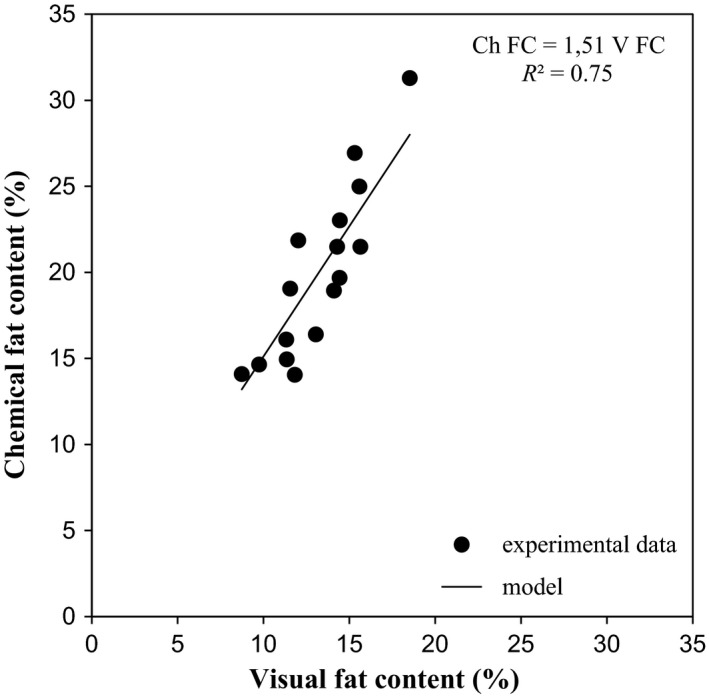
Comparison of fat content by chemical measurement (Ch FC) and image analysis (V FC) for sliced salami (data: *filled circles*; model: *continuous line*)

In order to analyze in details the relations between the image analysis parameters (size and shape features) and fat chemical contents of samples (Trials A–P), factorial PCA was applied to the experimental data. Figure 4a,b show plots of loadings and scores obtained from PCs, where the first two principal components (PC1 and PC2) accounted for 79% of the total variance of the data. In particular, the principal component PC1 explained 52% of the variation in the data, while the principal component PC2 explained 27%. The PC1 on the positive axis of the plane was positively correlated with all attributes, in particular AF_*i*max_, V FC and total fat amount (Ch FC) showed the highest eigenvalues (>0.9) on the first component (Figure 4a). Regarding PC2, the main positive contribution was due to $\bar{AF_{i}}$, while RF_*i*max_ and the total number of fat particles normalized by the total area of the sample slice (*n*/As) showed negative eigenvalues (Figure 4a). The samples were distributed in the all space defined by PCs (Figure 4b). In particular, C, F, M, N, K, positioned on the positive side of PC1, were associated with statistical parameters of area ($\bar{{\text{AF}}_{i}}$ and AF_*i*max_), $\bar{{\text{RF}}_{i}}$, and the visible and chemical fat content of sliced salami (V FC and Ch FC), while I, H, P, O were characterized by RF_*i*max_ and *n*/As. Another group, showing negative scores on the PC1 and located entirely in the positive part of PC2, was composed of B, while the last group, having negative scores on both PC1 and PC2, was represented by A, E, L. The results of PCA indicate that visual fat content is highly positively related to chemical fat composition of sliced salami. The correlation between the fat contents obtained with the two methods is shown in Figure 5. The performance of the image analysis protocol was positive and satisfactory (*R* ^2^=0.75), because results were comparable with the corresponding values obtained by the chemical analysis, with the advantage of information relating to the spatial distribution of fat particles.

## 4 CONCLUSIONS

The protocol of image analysis presented permits the automatic, accurate, objective and reliable quantification of visible fat particles of sliced ready‐to‐eat meat products such as salami.

The method does not require sophisticated equipment or advanced image analysis programs. The image analysis protocol extracts directly interpretable parameters of fat particle morphology (area, roundness) and number of fat particles Good relationships were found between the fat particle characteristics measured by digital vision and the percentage of chemically extractable fat particles by correlation and PCA analysis. Indeed, the results of PCA indicate the close relationship between image size and shape features and chemical composition of sliced salami.

The results confirmed that the image analysis protocol developed could extract the fat content automatically with good degree of accuracy (*R* ^2^=0.75), with the advantage of information relating to the spatial distribution of fat particles. The size distribution and shape of fat particles can in fact provide useful information to characterize and control meat products, such as Italian dry fermented sausages, which have a similar fat content, but a different size distribution of fat particles (e.g., some Protected Designation of Origin—PDO and Traditional Speciality Guaranteed—TSG meat products: “soppressata di Calabria”, “GIOI soppressata” and “salame Napoli”, etc.). Furthermore the images used by the protocol to determine fat content are capable of giving a wide range of additional information such as color (that is not the focus of our present work).

Further testing on larger type of meat products will be necessary before the presented procedure can be accepted and then used as a common method for measuring lipid in sliced sausage.

## 5 CONFLICT OF INTEREST

None declared.

## References

[fsn3540-bib-0001] AMSA (2001). Meat evaluation handbook. Savoy, IL: American Meat Science Association.

[fsn3540-bib-0002] AOAC (1991). Official methods of analysis (14th edn). Washington, DC: Association of Official Analytical Chemists.

[fsn3540-bib-0003] AOCS (1998). Method Ba 3–38 In Firestone D. (Ed.), Official methods and recommended practices of the American Oil Chemists Society (6th edn, 4pp). Champaign, IL: AOCS.

[fsn3540-bib-0004] Chmiel, M. , Słowiński, M. , & Dasiewicz, K. (2011). Lightness of the color measured by computer image analysis as a factor for assessing the quality of pork meat. Meat Science, 88, 566–570. 21392895 10.1016/j.meatsci.2011.02.014

[fsn3540-bib-0005] Conter, M. , Zanardi, E. , Ghidini, S. , Pennisi, L. , Vergara, A. , Campanini, G. , & Ianieri, A. (2007). Survey on typology, PRPs and HACCP plan in dry fermented sausage sector of Northern Italy. Food Control, 18, 650–655.

[fsn3540-bib-0006] Damez, J.‐L. , & Clerjon, S. (2008). Meat quality assessment using biophysical methods related to meat structure. Meat Science, 80, 132–149. 22063178 10.1016/j.meatsci.2008.05.039

[fsn3540-bib-0007] Dransfield, E. , Ngapo, T. M. , Nielsen, N. A. , Bredahl, L. , Sjodén, P. O. , Magnusson, M. , … Nute, G. R. (2005). Consumer choice and suggested price for pork as influenced by its appearance, taste and information concerning country of origin and organic production. Meat Science, 69, 61–70. 22062640 10.1016/j.meatsci.2004.06.006

[fsn3540-bib-0008] Du, C. J. , Sun, D. W. , Jackman, P. , & Allen, P. (2008). Development of a hybrid image processing algorithm for automatic evaluation of intramuscular fat content in beef M. longissimus dorsi. Meat Science, 80, 1231–1237. 22063863 10.1016/j.meatsci.2008.05.036

[fsn3540-bib-0009] Faucitano, L. , Huff, P. , Teuscher, F. , Gariepy, C. , & Wegner, J. (2005). Application of computer image analysis to measure pork marbling characteristics. Meat Science, 69, 537–543. 22062993 10.1016/j.meatsci.2004.09.010

[fsn3540-bib-0010] Ferguson, D. M. (2004). Objective on‐line assessment of marbling: A brief review. Australian Journal of Experimental Agriculture, 44, 681–685.

[fsn3540-bib-0011] Fernàndez, X. , Monin, G. , Talmant, A. , Mourot, J. , & Lebret, B. (1999). Influence of intramuscular fat content on the quality of pig meat. 1. Composition of the lipid fraction and sensory characteristics of m. longissimus lumborum. Meat Science, 53, 59–65. 22062933 10.1016/s0309-1740(99)00037-6

[fsn3540-bib-0500] Fongaro, L. , Alamprese, C. , Casiraghi, E. , (2015). Ripening of salami: Assessment of colour and aspect evolution using image analysis and multivariate image analysis. Meat Science, 101, 73–77. 25437453 10.1016/j.meatsci.2014.11.005

[fsn3540-bib-0012] Fortomaris, P. , Arsenos, G. , Georgiadis, M. , Banos, G. , Stamataris, C. , & Zygoyannis, D. (2006). Effect of meat appearance on consumer preferences for pork chops in Greece and Cyprus. Meat Science, 72, 688–696. 22061881 10.1016/j.meatsci.2005.09.019

[fsn3540-bib-0013] Girolami, A. , Napolitano, F. , Faraone, D. , Di Bello, G. , & Braghieri, A. (2014). Image analysis with the computer vision system and the consumer test in evaluating the appearance of Lucanian dry sausage. Meat Science, 96, 610–616. 24041911 10.1016/j.meatsci.2013.08.006

[fsn3540-bib-0014] Hur, S. J. , Park, G. B. , & Joo, S. T. (2008). A comparison of the meat qualities from the Hanwoo (Korean native cattle) and Holstein steer. Food and Bioprocess Technology, 1(2), 196–200.

[fsn3540-bib-0015] Jackman, P. , & Sun, D.‐W. (2013). Recent advances in image processing using image texture features for food quality assessment. Trends in Food Science & Technology, 29, 35–43.

[fsn3540-bib-0016] Kays, S. J. (1991). Postharvest physiology of perishable plant products. New York: Van Nostrand Reinholt.

[fsn3540-bib-0017] Mendoza, F. , Valous, N. A. , Sun, D.‐W. , & Allen, P. (2009). Characterization of fat‐connective tissue size distribution in pre‐sliced pork hams using multifractal analysis. Meat Science, 83, 713–722. 20416632 10.1016/j.meatsci.2009.08.009

[fsn3540-bib-0018] Mòrlein, D. , Rosner, F. , Brand, S. , Jenderka, K.‐V. , & Wicke, M. (2005). Non‐destructive estimation of intramuscular fat content of the longissimus muscle of pork by means of spectral analysis of ultrasound echo signals. Meat Science, 69, 187–199. 22062808 10.1016/j.meatsci.2004.06.011

[fsn3540-bib-0019] O'Sullivan, M. G. , Byrne, D. V. , Martens, H. , Gidskehaug, L. H. , Andersen, H. J. , & Martens, M. (2003). Evaluation of pork colour: Prediction of visual sensory quality of meat from instrumental and computer vision methods of colour analysis. Meat Science, 65(2), 909–918. 22063455 10.1016/S0309-1740(02)00298-X

[fsn3540-bib-0020] Penfield, M. P. , Costello, C. A. , McNeil, M. A. , & Rienmannn, M. J. (1989). Effects of fat level and cooking methods on physical and sensory characteristics of reestructure beef streaks. Journal of Food Quality, 11, 349–356.

[fsn3540-bib-0021] Romano, A. , Cavella, S. , Toraldo, G. , & Masi, P. (2013). 2D Structural imaging study of bubble evolution during leavening. Food Research International, 50, 324–329.

[fsn3540-bib-0022] Serrano, S. , Perán, F. , Jiménez‐Hornero, F. J. , & Gutiérrez de Ravé, E. (2013). Multifractal analysis application to the characterization of fatty infiltration in Iberian, and White pork sirloins. Meat Science, 93, 723–732. 23247059 10.1016/j.meatsci.2012.11.015

[fsn3540-bib-0023] Sørheim, O. , Måge, I. , & Larsen, H. (2017). Determination of critical levels of residual oxygen to minimize discoloration of sliced packaged Norwegian salami under light display. Meat Science, 129, 88–92. 28267645 10.1016/j.meatsci.2017.02.014

[fsn3540-bib-0024] Tan, J. (2004). Meat quality evaluation by computer vision. Journal of Food Engineering, 61(1), 27–35.

[fsn3540-bib-0025] Touraine, B. , Vigneror, P. , Touraille, C. , & Touraille, M. (1984). Influence des conditions d'elevage sur les characteristiques des carcasses et de la viande d'agneaux. Bulletin Technique de l'Elevage Ovin, 4, 29–40.

[fsn3540-bib-0026] Wu, D. , & Sun, D.‐W. (2013). Advanced applications of hyperspectral imaging technology for food quality and safety analysis and assessment: A review — Part II: Applications. Innovative Food Science and Emerging Technologies, 19, 15–28.

